# Novel Push-Pull Benzodithiophene-Containing Polymers as Hole-Transport Materials for Efficient Perovskite Solar Cells

**DOI:** 10.3390/molecules27238333

**Published:** 2022-11-29

**Authors:** Aleksandra N. Mikheeva, Ilya E. Kuznetsov, Marina M. Tepliakova, Aly Elakshar, Mikhail V. Gapanovich, Yuri G. Gladush, Evgenia O. Perepelitsina, Maxim E. Sideltsev, Azaliia F. Akhkiamova, Alexey A. Piryazev, Albert G. Nasibulin, Alexander V. Akkuratov

**Affiliations:** 1 Federal Research Center of Problems of Chemical Physics and Medicinal Chemistry, Russian Academy of Sciences, FRC PCPMC RAS, Academician Semenov Avenue 1, 142432 Chernogolovka, Russia; 2 Skolkovo Institute of Science and Technology, Nobel St. 3, 143026 Moscow, Russia; 3 Departament of Chemistry, Lomonosov Moscow State University, GSP-1, 1 Leninskiye Gory, 119991 Moscow, Russia; 4 Sirius University of Science and Technology, 1 Olympic Ave., 354340 Sochi, Russia

**Keywords:** benzodithiophene, conjugated polymers, hole-transport materials, perovskite solar cells, film-forming ability

## Abstract

Donor-acceptor conjugated polymers are considered advanced semiconductor materials for the development of thin-film electronics. One of the most attractive families of polymeric semiconductors in terms of photovoltaic applications are benzodithiophene-based polymers owing to their highly tunable electronic and physicochemical properties, and readily scalable production. In this work, we report the synthesis of three novel push–pull benzodithiophene-based polymers with different side chains and their investigation as hole transport materials (HTM) in perovskite solar cells (PSCs). It is shown that polymer **P3** that contains triisopropylsilyl side groups exhibits better film-forming ability that, along with high hole mobilities, results in increased characteristics of PSCs. Encouraging a power conversion efficiency (PCE) of 17.4% was achieved for **P3**-based PSCs that outperformed the efficiency of devices based on **P1**, **P2**, and benchmark PTAA polymer. These findings feature the great potential of benzodithiophene-based conjugated polymers as dopant-free HTMs for the fabrication of efficient perovskite solar cells.

## 1. Introduction

Perovskite solar cells (PSCs) are cost-effective and rapidly advancing photovoltaic technology showing great potential to compete with silicon solar cells, as well as to achieve strong positions on the market. Optimization of device structure and exploration of new absorber materials resulted in an unprecedentedly rapid increase in power conversion efficiency from ca. 4% to more than 25% [1,2]. Hole-transporting materials (HTMs) also play a crucial role in boosting performance and improving the operational stability of PSCs. In particular, individual or double-layered HTMs based on a combination of metal oxides and organic polymers can prevent the migration of the volatile byproducts of perovskite reversible decomposition from the device, so they could participate in the perovskite formation reaction enabling improved operation stability of PSCs [3,4]. Many various organic HTMs have recently been developed and investigated in PSCs. Among the most promising HTMs, the Spiro-OMeTAD (2,2′,7,7′-tetrakis(N,N-di-p-methoxyphenyl-amine)9,9′-spirobifluorene) [5,6] and PTAA (poly[bis(4-phenyl)(2,4,6-trimethylphenyl)amine]) [7] are widely used. Unfortunately, these benchmark HTMs provide the impressive performance of PSCs only after doping, which usually suggests use of hygroscopic lithium salts coupled with further oxidation, thus leading to device stability mitigation [8]. In sum, the development of dopant-free, cost-effective HTMs with tunable electronic properties—high hole mobilities is of great importance. Moreover, good film formation properties of HTMs and uniform morphology of films are substantial aspects for the fabrication of high-performance PSCs [9,10]. In this regard, benzo[1,2-b:4,5-b′]dithiophene-based (BDT) conjugated polymers can be considered as prospective HTMs. BDT is a fused tricyclic building block with a rigid planar structure and extended π–electron-delocalization, which facilitates efficient charge transport in BDT-based conjugated polymers [11]. Moreover, the BDT core can be easily functionalized by heteroaryl and alkyl groups in 4 and 8 positions, which also enables adjustment of the energy levels and morphology, which are important factors for improving the charge extraction and transport. Additionally, the coverage of perovskite with hydrophobic organic polymers enable the protection of the photoactive layer from moisture ingress.

**Figure 1:**
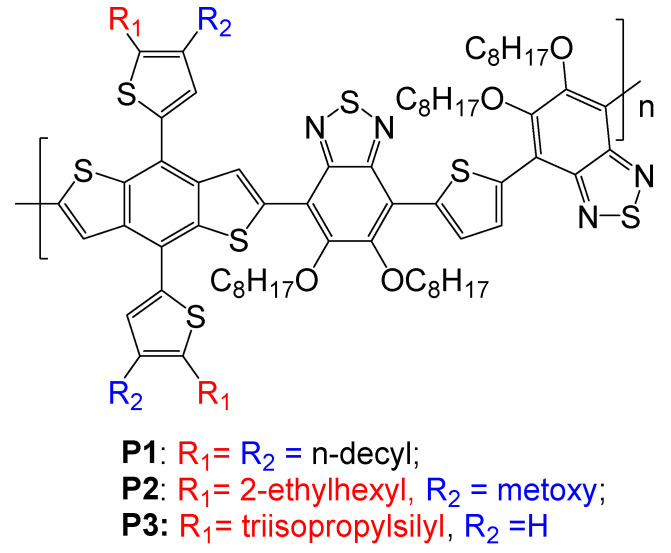
Conjugated polymers **P1**–**P3**.

Here, we report the synthesis of three novel BDT-based conjugated polymers and the investigation of these compounds, such as HTMs in PSCs. The polymers **P1**–**P3** were designed according to the push–pull concept by combining BDT moieties bearing different alkyl side chains with benzothiadiazole acceptor and thiophene donor moieties to ensure favorable electronic and physicochemical characteristics, e.g., deep HOMO energy level, high charge mobilities, good solubility, and film formation properties (Figure 1).

Variation of side chains on BDT core allowed additionally to investigate the effect of HTMs structure on their physico-chemical properties and charge-transport characteristics, which are important prerequisites for high performance of PSCs based on them.

## 2. Results and Discussion

**Figure 2:**
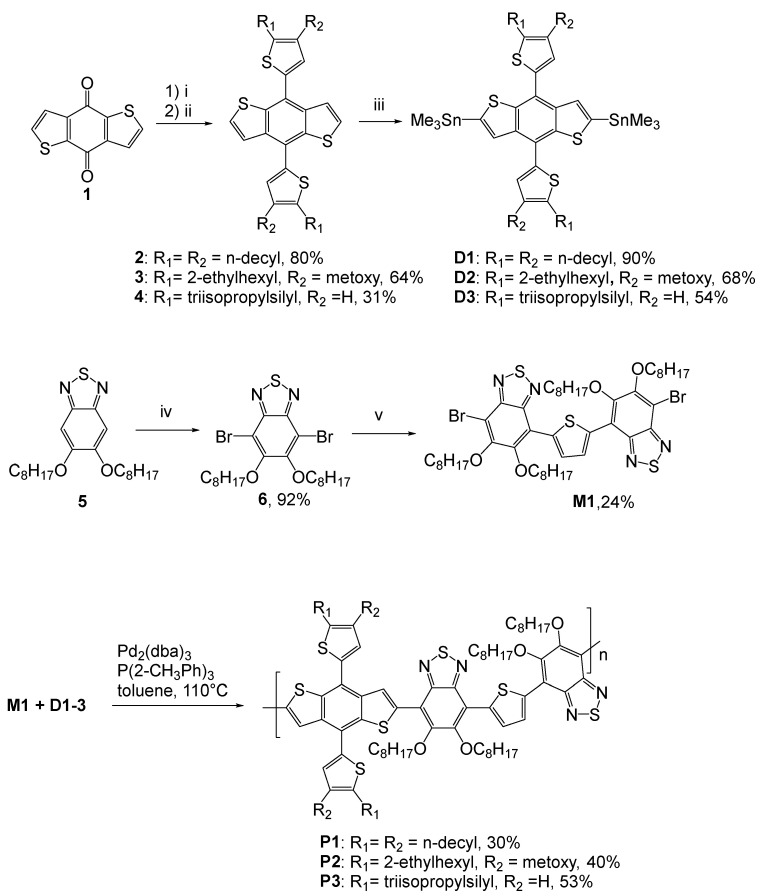
Synthesis of monomers **D1**–**D3**, **M1** and conjugated polymers **P1**–**P3**. Conditions: i—(4,5-didecylthien-2-yl)lithium or (4-(2-ethylhexyl)-5-(methoxy)thien-2-yl)lithium or (5-(triisopropylsilyl)thien-2-yl)lithium, THF; ii—SnCl_2_•2H_2_O, 10% HCl; iii—BuLi, −78 °C, THF, TMEDA, SnMe_3_Cl; iv—Br_2_, AcOH, 60 °C; v—2,5-bis(trimethylstannyl)thiophene, toluene, 110 °C, Pd(PPh_3_)_4_.

The synthetic route for the preparation of benzodithiophene-based monomers **D1**–**D3**, benzothiadiazole-thiophene monomer **M1** and conjugated polymers **P1**–**P3** is shown in Figure 2. The reaction of benzo[1,2-b:4,5-b′]dithiophene-4,8-dione (**1**) [12] with (4,5-dialkylthien-2-yl)lithium derivatives was followed by reduction of the corresponding diols with tin (II) chloride in hydrochloric acid afforded compounds **2**–**4**. The monomers **D1**–**D3** were obtained by lithiation of benzo[1,2-b:4,5-b′]dithiophenes **2**–**4** with *n*-BuLi and quenching of lithium derivatives with trimethyltin chloride.

Monomer **M1** was synthesized in three steps. First, 5,6-bis(octyloxy)-2,1,3-benzothiadiazole [13] (**5**) was brominated with bromine in acetic acid. Further palladium-catalyzed Stille coupling of compound **6** (1 eq., 1.82 mmol) and 2,5-bis(trimethylstannyl)thiophene (0.2 eq., 0.36 mmol) delivered monomer **M1**. Conjugated polymers **P1**–**P3** were synthesized via Stille polycondensation reaction.

Polymers **P1**–**P3** were precipitated with methanol, dried under reduced pressure, and then washed in Soxhlet apparatus with acetone, hexane, and chloroform. The chloroform fractions were concentrated on a rotary evaporator and an excess of methanol was added. The formed precipitates were collected by filtration, followed by drying in a nitrogen atmosphere (10^−2^ mm Hg). Relative molecular weight characteristics of conjugated polymers were determined by gel-permeation chromatography (GPC) against polystyrene standards (Figure S1). The obtained weight-average molecular weights of polymers **P1**–**P3** are in the range of 13–22 kDa (Table 1). All polymers have good solubility in chloroform, toluene, and chlorobenzene at room temperature, which makes them suitable for deposition using solution-based techniques. The polymers were characterized by NMR spectroscopy (Figures S2–S4) and FTIR spectroscopy (Figure S5).

**Figure 3:**
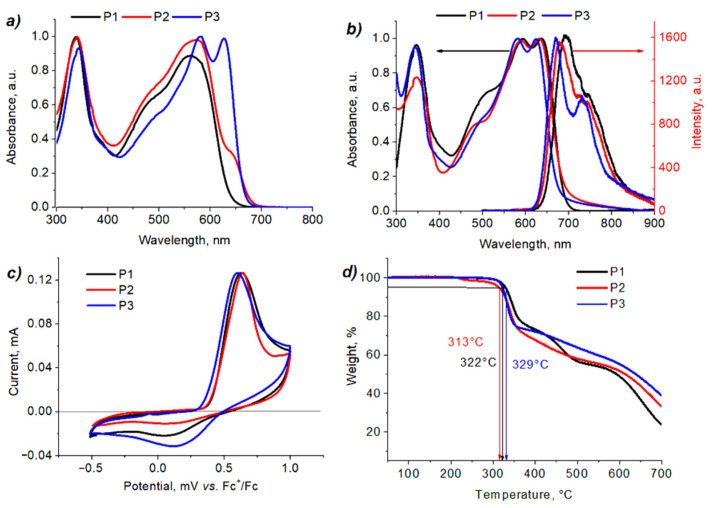
Absorption spectra of polymers **P1**–**P3** in 1,2-dichlorobenzene solution (**a**); absorption and emission spectra of thin films (**b**); cyclic voltammograms of polymer thin films (supporting electrolyte—0.1 M Bu_4_NPF_6_ in acetonitrile, scan rate—50 mVs^−1^, glassy carbon disc electrode) (**c**); TGA curves of polymers **P1**–**P3** (**d**).

Thermal properties of polymers were investigated by thermal gravimetric analysis (TGA) and differential scanning calorimetry (DSC) under an inert atmosphere. The 5% weight loss, which corresponds to the decomposition of materials, was observed at high temperatures from 313 °C to 329 °C for all materials (Figure 3d). Multistep destruction of macromolecules can probably be attributed to the scission of thermolabile octyloxy side chains followed by the destruction of the polymer backbone.

No signals on the DSC curves were observed for polymers **P2** and **P3**, which reveals the amorphous nature of materials in the solid state. Heating and cooling the DSC curves for polymer **P1** showed low-intensity broad flat peaks with a T_m_ of 158.8 °C and 189.7 °C, and T_c_ of 163.9 °C (Figure S6). This implies certain self-organization of polymer **P1** in the solid state, that might be attributed to the presence of four linear decyl side chains on thiophene fragments, which enhance non-covalent intermolecular interactions [14]. The XRD patterns of thin films of polymers **P1**–**P3** revealed peaks at 2θ = 4–5°, which correspond to the short-range order, while the broad signal in the range of 2θ = 20–25° is typical for interactions via π–π stacking (Figure S7).

Optoelectronic properties of polymers were investigated by absorption spectroscopy, photoluminescence spectroscopy and cyclic voltammetry. All polymers exhibited two main absorption bands at 300–400 nm and 450–700 nm, which are attributed to π–π* transitions and intramolecular charge transfer from donor to acceptor moieties, respectively (Figure 3).

In 1,2-dichlorobenzene solution, conjugated polymers demonstrated quite different optical properties, while in thin film spectra, $\lambda_{max\;}^{film}$ and $\lambda_{edge\;}^{film}$ are very similar. When comparing solution and film spectra (Figure S8), the most pronounced bathochromic shifts can be seen for absorption bands of **P1** and **P2** that may be due to strong self-aggregation of these polymers in the solid state. The minor red shift of the absorption band in the case of **P3** suggests stronger aggregation of macromolecules even in solution at room temperature that was supported by temperature-dependent UV-visible spectra (Figure S8).

The emission bands in the photoluminescence spectra (PL) of polymers are shifted in a similar manner as in absorption spectra. The emission maxima for **P1**, **P2**, and **P3** are located at 693 nm, 678 nm, and 670 nm, respectively. The Stokes shifts are in the range of 35.4–44.4 meV, which points to little structural difference between the excited and ground states of macromolecules. The lowest Stoke shift of 35.4 meV for **P3** reveals insignificant reorganization energy of the charge carriers (polarons), which limits the intrinsic mobility of organic semiconductors [15,16]. Additionally, the most pronounced vibronic peak in the PL spectrum for **P3** at 731 nm suggests a rigid character of macromolecules in the ground state. The optical bandgap of polymers was estimated as E_g_ = 1240/λ_int_, where λ_int_ is the wavelength at the intersection of corresponding absorption and emission bands. The E_g_ values of 1.84 eV, 1.85 eV, and 1.86 eV were found for **P1**, **P2**, and **P3**, respectively. The HOMO energy levels of the polymers were determined from the oxidation onsets (Figure 3c) as $HOMO=-(E_{onset\;}^{ox}$ + 4.8) eV [17,18]. The onsets of oxidation potentials for **P1**, **P2**, and **P3**, were found to be 0.40 V, 0.39 V, and 0.36 V, corresponding to the HOMO energies of −5.20 eV, −5.19 eV, and −5.16 eV, respectively. Thus, the variation of side chains in the polymer backbone does not significantly affect the energy of frontier orbitals and energy bandgap (Table 1).

The matching of frontier orbital energies with a valence band (VB) and conductive band (CB) of perovskite is important for the selective extraction of one type of charge carriers (holes or electrons) and blocking of opposite charges in order to reduce the recombination processes. Since the HOMO and LUMO energies of polymers are well aligned with VB = −5.43 eV and CB = −3.93 eV of perovskite absorber (in this work, CH_3_NH_3_PbI_3_ or MAPbI_3_), these compounds can be used as hole transport materials in perovskite solar cells [19].

The polymers **P1**–**P3** were evaluated as HTM in PSCs with n-i-p architecture ITO/SnO_2_/[6,6]-phenyl-C_61_-butyric acid (PCBA)/MAPbI_3_/**P1**–**P3**/MoO_3_/Ag. Thin films of conjugated polymers **P1**–**P3** were spin-coated from chlorobenzene solutions on top of the perovskite layer using preliminary optimized deposition conditions, and **PTAA** was used as a reference HTM. The detailed procedure was reported previously [20].

**Figure 4:**
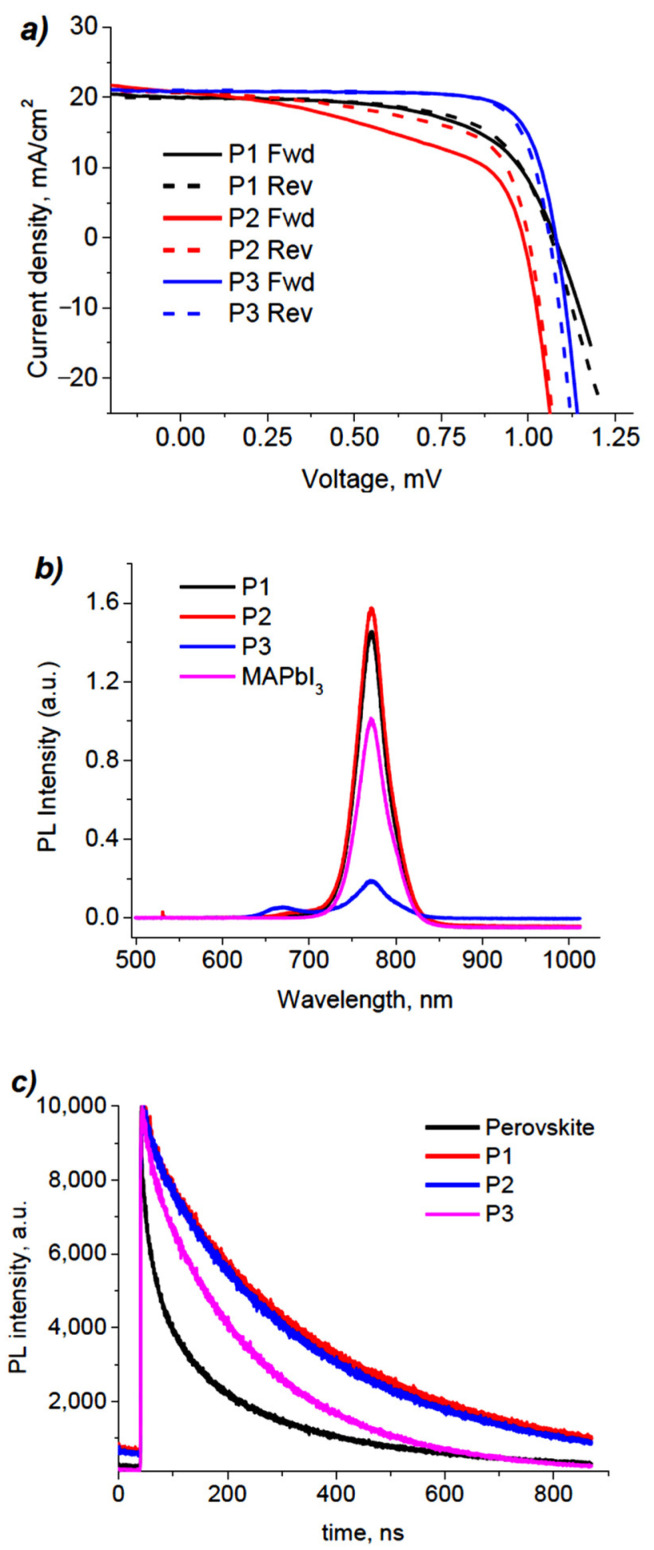
*J*-V curves of PSCs fabricated under optimized conditions (**a**); steady-state PL spectra for glass/perovskite/HTM stacks (**b**); time-resolved PL measurements of the perovskite films deposited on the **P1**–**P3** HTMs (**c**).

The photovoltaic characteristics of PSCs were investigated under AM 1.5 G (100 mW cm^−2^) illumination. The highest PCE of 17.4% was obtained for PSCs with polymer **P3** outperforming that with **PTAA** (PCE = 15.7%), **P1** (PCE = 13.7%), and **P2** (PCE = 12.4%), which is an encouraging result considering that HTM layer was processed without any kind of doping (Figure 4a, Table 2). Higher *J*_SC_ values of 20.9 mA cm^−2^ and FFs of 77% suggest that **P3** possess sufficient charge transport properties. On the contrary, solar cells with polymers **P1** and **P2** exhibited lower fill factors, causing a remarkable decrease in efficiency. Additionally, forward and reverse scans of **P2**-based PSCs demonstrated noticeable hysteresis. This effect can result from inferior hole-transport properties or the hole-extraction ability of polymers **P1** and **P2** in contrast to **P3**, making it more suitable for HTM application [21].

Next, we estimated the hole mobilities of HTMs by a space-charge-limited current (SCLC) technique. Hole mobilities were found to be 3.63 × 10^−5^ cm^2^V^−1^s^−1^, 2.89 × 10^−5^ cm^2^V^−1^s^−1^, and 4.10 × 10^−5^ cm^2^V^−1^s^−1^ for **P1**, **P2**, and **P3**, respectively. The higher hole mobility for **P3** can be attributed to the better molecular packing due to improved interchain interactions arising from σ*(Si)-π*(C) bond interactions that are in agreement with previously reported results [22,23]. Thus, enhanced characteristics of **P3**-based perovskite solar cells can be explained by higher hole mobilities of the HTM films.

In order to characterize the hole-extracting ability provided by the materials, we recorded steady-state photoluminescence (PL) spectra of samples of glass/perovskite/HTM and glass/perovskite as a reference (Figure 4b). Samples with **P3** exhibited significant quenching of the PL intensity, which is usually attributed to the high hole extraction ability of the material. The signal observed at 660 nm corresponds to the intrinsic PL of **P3**. Surprisingly, the PL intensities of MAPbI_3_/**P1** and MAPbI_3_/**P2** bilayers were higher than that of bare perovskite film. Such an increase in the PL intensity may be the result of the passivation of trap states on the surface of perovskite and, therefore, mitigation of non-radiative recombination [24,25]. This conclusion is consistent with time-resolved PL measurements (Figure 4c). The effect is more pronounced in the case of **P1** and **P2** in accordance with steady-state PL. The exponential fitting of signal decay revealed the shortest charge carrier lifetime (τ_1_ = 191.4 ns) for the sample with **P3**, indicating a more efficient extraction of holes provided by this material [26,27].

Scanning electron microscopy (SEM) and atomic force microscopy (AFM) were used to explore the differences in the surface appearances of perovskite/**P1**–**P3** samples (Figure S9). Both top-view SEM and AFM images revealed uniform and homogenous surface morphology, which means that the differences can not be found for freshly prepared devices on the microscale.

**Figure 5:**
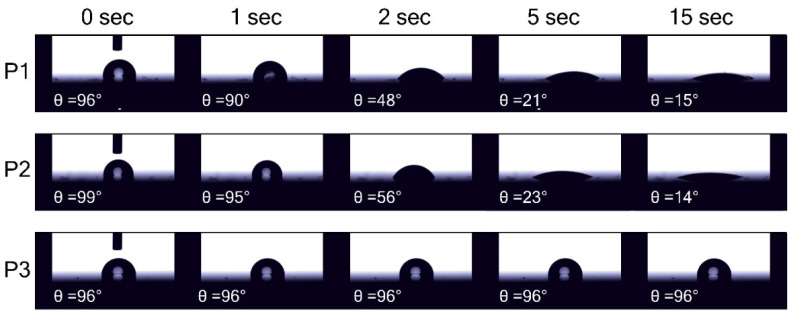
Changes of contact angle of water on perovskite/HTM sample.

To evaluate the hydrophobicity and quality of coverage provided by polymers, we measured further the contact angle (θ) of water on the surface of **P1**–**P3** films deposited on perovskite (Figure 5). For all samples, θ = 96–99° at the initial moment suggested the hydrophobic nature of the films. It can be seen that θ values for **P3** film did not change with time, while for **P1** and **P2**-based samples, the θ values rapidly decreased, indicating the interaction of water with the more hydrophilic perovskite layer. The measurements of contact angles on glass/polymer substrates showed similar results, with θ = 32–38° for **P1** and **P2**, and θ≈107° for **P3** (Figure S10). Optical microscopy examination of the substrates allowed us to note the circles, which appeared due to the reconstruction of the polymer layer after water deposition. In this case, the polymer film reconstruction facilitates contact between water and glass (Figure S11a). At the same time, neither defects nor similar circles form on the perovskite/**P3** layer. To explore further polymer film reconstruction, the microphotographs of the samples glass/perovskite/**P1**–**P3** just after water drop deposition were collected (Figure S11b). The spots on the surface of **P1** and **P2**-based samples exposed to water are separated from undamaged perovskite by the rather thick boundary similar to the ones observed for perovskite-free samples. As for **P3,** there is a comparatively small area where perovskite is in contact with water and no similar boundaries appeared. Thus, we assume that the adhesion of polymer **P3** to perovskite is strong enough to prevent its delamination and parting.

It is worth noting that in the devices, the layer deposited atop the polymer is inherently polar metal oxide (MoO_3_). In the case of polymers **P1** and **P2**, the layer deformation after MoO_3_ deposition may occur, leading to defects on HTM/perovskite interface and, thus, to inferior performance of PSCs. Therefore, better adhesion of **P3** films to perovskite probably enabled by a higher molecular weight of the polymer, resulting in the better quality of films and hence, improved performance of PSCs.

Thus, encouraging PCEs of 17.4% was achieved for devices with **P3** as HTM without any doping. We envision that the double-layered polymer films such as **P1**/**P3** or **P2**/**P3** might be promising HTMs, due to their ability for passivation of perovskite defects along with good charge transport characteristics. The presented results provide good insights into the role of molecular engineering on the physicochemical properties of novel BDT-based HTMs and their performance in perovskite solar cells.

## 3. Materials and Methods

All solvents and starting compounds as benzo[1,2-b:4,5-b′]dithiophene-4,8-dione (CAS Number 32281-36-0), 2,5-bis(trimethylstannyl)thiophene (CAS Number: 86134-26-1), were purchased from Sigma-Aldrich (Saint Louis, MO, USA) or Acros Organics (Geel, Belgium) and used as received.

Molecular weight distribution was analyzed using gel-permeation chromatography (GPC) using Waters GPCV 2000 chromatograph (column PL-gel, 5 µm, MIXED-C, 300 × 7.5 mm) equipped with a refractometer. Tetrahydrofuran was used as an eluent. All measurements were carried out at 40 °C; the flow rate was 1 mL/min. “EMPOWER,” and Astra 5.3.2.20 software was used for data processing.

FT-IR spectra were recorded in the 400–4000 cm^−1^ range (48 scans, resolution 4 cm^−1^) using a Bruker ALPHA.

Absorption spectra for dilute solutions of polymers (1 × 10^−4^ M) and thin films deposited by spin-coating from 1 × 10^−2^ M solutions on quartz substrates were measured in UV-visible region (300–800 nm) on scanning spectrophotometer SPECS SSP-705-1.

The ^1^H and ^13^C NMR spectra were obtained using Bruker AVANCE 500 instrument. The AFM images were obtained using an NTEGRA PRIMA instrument (NT-MDT, Russia).

The cyclic voltammetry measurements were performed for thin films of the polymers **P1**–**P3** deposited on a glassy carbon disc electrode by drop casting from chlorobenzene. The measurements were performed in a three-electrode electrochemical cell using a 0.1 M solution of Bu_4_NBF_4_ in acetonitrile as a supporting electrolyte, platinum wire as a counter electrode, and Ag/Ag^+^ (in 0.01 M acetonitrile) as a reference electrode. Ferrocene was used as an internal reference. The voltammograms were recorded using an ELINS P-20-X instrument at room temperature in the range −0.5–1.0 V with a potential sweep rate of 50 mV s^−1^ [28].

The thermal properties of the polymers were investigated by thermal gravimetry analysis (TGA) using Q50 TA instruments under nitrogen with a heating rate of 10 °C min^−1^. Differential scanning calorimetry (DSC) was performed using a Netzsch DSC 214 Polyma instrument in the same conditions.

The *J*-V curves were recorded in the glovebox under the illumination (100 mW/cm^2^) provided by Newport Verasol AAA solar simulator using Advantest 6240A source-measurement units.

*Fabrication of solar cells with* **P1–P3** *for the efficiency investigation*

The glass substrates covered with indium tin oxide (ITO, Kintec, 15 Ω/sq.) were preliminarily treated with ultrasonication in acetone, water, isopropanol and air-plasma (5 min). The 10% suspension of SnO_2_ nanoparticles in water (Alfa Aesar, Kandel, Germany) was spin-coated at 4000 rpm in two steps for 20 sec. Further, samples were placed in the cold heater and heated up to 165 °C for 10 min and annealed at this temperature for 30 min. The passivation layer for SnO_2_ (PCBA, 0.2 mg/mL in chlorobenzene (CB)) was spin-coated in the glovebox. The samples were annealed at 100 °C for 10 min. The solution of MAPbI_3_ perovskite (1.4M) was prepared by dissolving equimolar amounts of methylammonium iodide and lead iodide in the mixture of dimethylformamide (80%) and n-methylpyrrolidone (20%) to give a 1.4M solution of the perovskite ink. Perovskite was spin-coated at 3000 rpm dynamically and left to dry for 20 min. In the next step, samples were heated up to 80 °C for 10 min and annealed at this temperature for the next 5 min. Solutions of polymers (6 mg/mL in CB) were spin-coated at 2000 rpm, which was preliminarily confirmed as the optimal deposition condition for each material. The layer of MoO_x_ (10 nm) was thermally evaporated on the full area (under 10^−5^ mbar). Finally, the Ag top electrode (90–100 nm) was deposited using thermal evaporation through the shadow mask, defining the area of the final cells to be 0.1 cm^2^.

*Synthesis of compounds*

Starting compounds **1a** [29], **1b** [30], **1c** [31], **5** [32] were synthesized by previously reported methods.

*Synthesis of compound* **2**

Compound **2** was prepared using 2,3-didecylthiophene (**1a**) (5 g, 13.7 mmol), n-BuLi 2.5 M in hexane (5.48 mL, 13.7 mmol), and benzo[1,2-b:4,5-b]dithiophene-4,8-dione (1.58 g, 6.85 mmol). Then SnCl_2_∙2H_2_O (6.2 g, 27.4 mmol) in 60 mL of 10% HCl was added and the compound was isolated and purified as reported previously [29]. Yield: 80% ^1^H NMR (CDCl_3_, 500 MHz, δ): 7.68 (d, 2H), 7.43 (d, 2H), 7.21 (s, 2H), 2.81 (t, 4H), 2.60 (t, 4H), 1.75–1.62 (m, 8H), 1.28 (m, 56H), 0.88 (m, 12H) ppm. ^13^C NMR (126 MHz, CDCl_3_, δ): 140.17, 138.86, 138.09, 136.36, 135.08, 129.84, 127.22, 124.14, 123.56, 31.91, 31.82, 30.79, 29.65, 29.63, 29.61, 29.55, 29.49, 29.43, 29.34, 28.31, 28.00, 22.67, 14.07.

*Synthesis of compound* **3**

Compound **3** was prepared using 2-(2-ethylhexyl)-3-methoxythiophene (**1b**) (3.1 g, 13.7 mmol), n-BuLi 2.5M in hexane (5.48 mL, 13.7 mmol), and benzo[1,2-b:4,5-b]dithiophene-4,8-dione (1.58 g, 6.85 mmol). Then SnCl_2_∙2H_2_O (6.2 g, 27.4 mmol) in 60 mL of 10% HCl was added and the compound was isolated and purified as reported previously [30]. Yield: 64% ^1^H NMR (500 MHz, CDCl_3_, δ): 7.69 (d, 2H), 7.46 (d, 2H), 7.23 (s, 2H), 3.89 (s, 6H), 2.76 (d, 4H), 1.66 (m, 2H), 1.49–1.25 (m, 16H), 0.98–0.88 (m, 12H) ppm. ^13^C NMR (126 MHz, CDCl_3_, δ): 153.92, 139.09, 136.54, 134.13, 127.79, 124.39, 123.60, 123.01, 117.81, 59.34, 41.17, 32.79, 29.93, 29.11, 26.07, 23.28, 14.38, 11.11

*Synthesis of compound* **4**

Compound **4** was prepared using **1c** (triisopropyl(thiophen-2-yl)silane) (3.3 g, 13.7 mmol), n-BuLi 2.5M in hexane (5.48 mL, 13.7 mmol), and benzo[1,2-b:4,5-b]dithiophene-4,8-dione (1.58 g, 6.85 mmol). Then SnCl_2_∙2H_2_O (6.2 g, 27.4 mmol) in 60 mL of 10% HCl was added and the compound was isolated and purified as reported previously [30]. Yield: 31% ^1^H NMR (500 MHz, CDCl_3_, δ): 7.69 (d, 2H), 7.63 (d, 2H), 7.51 (d, 2H), 7.44 (d, 2H), 1.46 (m, 6H), 1.23 (d, 36H) ppm. ^13^C NMR (126 MHz, CDCl_3_, δ): 145.29, 143.30, 142.39, 138.71, 137.76, 131.23, 128.83, 127.45, 123.52, 18.69, 11.89.

*Synthesis of compound* **D1**

Compound **D1** was prepared using compound **2** (2.7 g, 3.1 mmol), 2.5 M n-BuLi (3.1 mL, 7.8 mmol), TMEDA (1.86 mL, 12.4 mmol), and trimethyltin chloride (1.55 g, 7.8 mmol) according to previously reported methods [33]. Yield: 90% ^1^H NMR (500 MHz, CDCl_3_, δ): 7.72 (s, 2H), 7.23 (s, 2H), 2.83 (t, 4H), 2.61 (t, 4H), 1.65–1.75 (m, 8H), 1.25–1.23 (m, 56H), 0.9–0.8 (m, 12H), 0.45–0.35 (m, 18H) ppm. ^13^C NMR (126 MHz, CDCl_3_, δ): 143.10, 141.91, 139.82, 138.00, 137.18, 135.77, 131.43, 129.83, 122.50, 31.94, 31.86, 30.86, 29.73, 29.70, 29.67, 29.55, 29.38, 28.35, 28.06, 22.71, 14.13, −8.34 ppm.

*Synthesis of compound* **D2**

Compound **D2** was synthesized and purified following the method described for **D1** using compound **3** (1.98 g, 3.1 mmol), 2.5 M n-BuLi (3.1 mL, 7.8 mmol), TMEDA (1.86 mL, 12.4 mmol), and trimethyltin chloride (1.55 g, 7.8 mmol) Yield: 68% ^1^H NMR (500 MHz, CDCl_3_, δ): 7.73 (s, 2H), 7.25 (s, 2H), 3.92 (s, 6H), 2.78 (d, 4H), 1.68 (m, 2H), 1.50–1.25 (m, 16H), 1.00–0.88 (m, 12H), 0.45–0.40 (m, 18H) ppm. ^13^C NMR (126 MHz, CDCl_3_, δ): 153.65, 143.17, 142.45, 137.19, 134.69, 131.17 122.54, 122.50, 117.54, 59.19, 40.96, 32.62, 29.82, 28.95, 25.96, 23.11, 14.21, 10.98, −8.33 ppm.

*Synthesis of compound* **D3**

Compound **D3** was synthesized and purified following the method described for **D1** using compound **5** (2.07 g, 3.1 mmol), 2.5 M n-BuLi (3.1 mL, 7.8 mmol), TMEDA (1.86 mL, 12.4 mmol), and trimethyltin chloride (1.55 g, 7.8 mmol) Yield: 54% ^1^H NMR (500 MHz, CDCl_3_, δ): 7.77 (s, 2H), 7.67 (d, 2H), 7.43 (d, 2H), 1.45 (m, 6H), 1.22 (d, 36H), 0.42 (s, 18H) ppm. ^13^C NMR (126 MHz, CDCl_3_, δ): 145.70, 143.06, 142.23, 137.26, 135.78, 135.09, 131.36, 128.64, 122.27, 18.69, 11.89, −8.41 ppm.

*Synthesis of compound* **6**

Compound **6** was prepared using compound **5** (1 g, 2.55 mmol) and *N*-bromosuccinimide (0.703 g, 5.1 mmol) according to previously reported methods [34]. Yield: 92% ^1^H NMR (CDCl_3_, 600 MHz, δ): 4.20 (t, 4H), 1.94–1.91 (m, 4H), 1.57–1.55 (m, 4H), 1.42–1.34 (m, 16H), 0.93 (t, 6H) ppm.

*Synthesis of compound* **M1**

Compound **M1** was prepared using **6** (1.0 g, 1.82 mmol), 2,5-bis(trimethylstannyl)thiophene (0.15 g, 0.36 mmol), toluene (50 mL) and tetrakis(triphenylphosphine)palladium(0) (10 mg, 0.47%mol) according to previously reported methods [33]. Yield: 24% ^1^H NMR (CDCl_3_, 500 MHz, δ): 8.51 (s, 2H), 4.20 (t, 4H), 4.12 (t, 4H), 1.92 (m, 4H), 1.55 (m, 4H), 1.43–1.21 (m, 40H), 0.88 (t, 6H), 0.81 (t, 6H) ppm.

*Synthesis of polymers* **P1–P3**

The polymers **P1**–**P3** were synthesized using toluene as solvent, Pd_2_(dba)_3_ (4 mg, 1.5%mol) as the catalyst and (2-CH_3_Ph)_3_P (2 mg, 0.0066 mmol) as a ligand. When the polymers did not show further increase in molecular weight within 2–3 h, the reaction was stopped by adding of excess of 2-trimethyl(2-thienyl)stannane and after a 1 h excess of 2-bromothiophene. The general procedure of synthesis and purification has been reported previously [35].

*Polymer* **P1**

Polymer **P1** was synthesized from **M1** (0.30 g, 0.29 mmol) and **D1** (0.364 g, 0.29 mmol) yield: 30%. M_w_ = 13.3 kDa, M_w_/M_n_ = 2.3. ^1^H NMR (CDCl_3_, 500 MHz, δ): 9.27 (s); 8.65 (m); 7.45 (s); 4.20 (m, br); 2.88 (s); 2.67 (s); 2.04–2.02 (m, br); 1.48–1.24 (m, br); 0.89–0.82 (m,br) ppm. FT-IR (KBr, ν): 3430, 2931, 2851, 1631, 1569, 1462, 1426, 1373, 1284, 1221, 1177, 1079, 1026, 954, 892, 847, 812, 723, 527 cm^−1^.

*Polymer* **P2**

Polymer **P2** was synthesized from **M1** (0.30 g, 0.29 mmol) and **D2** (0.283 g, 0.29 mmol) yield: 40%. M_w_ = 14.7 kDa, M_w_/M_n_ = 2.3. ^1^H NMR (CDCl_3_, 500 MHz, δ): 9.27 (s); 8.65 (m); 7.45 (s); 4.19 (m); 2.82 (s, br); 2.33 (m, br); 2.05–1.99 (m, br); 1.49–1.23 (m, br), 0.87–0.85 (m, br) ppm FT-IR (KBr, ν): 3436, 2925, 2855, 1767, 1625, 1563, 1465, 1429, 1376, 1278, 1206, 1171, 1082, 1028, 957, 895, 815, 726, 530 cm^−1^.

*Polymer* **P3**

Polymer **P3** was synthesized from **M1** (0.30 g, 0.29 mmol) and **D3** (0.291 g, 0.29 mmol) yield: 53%. M_w_ = 21.6 kDa, M_w_/M_n_ = 1.8. ^1^H NMR (CDCl_3_, 500 MHz, δ): 9.63 (m, br); 8.39–8.16 (m, br); 7.72 (d, br); 7.49 (m, br); 4.30–4.16 (m, br); 2.33–1.98 (m, br); 1.57–1.24 (m, br); 0.95–0.85 (m, br) ppm. FT-IR (KBr, ν): 3441, 2925, 2854, 1643, 1563, 1465, 1420, 1367, 1286, 1206, 1180, 1019, 966, 886, 806, 734, 681, 654, 565, 521 cm^−1^.

## 4. Conclusions

In this work, we synthesized three novel benzodithiophene-based polymers with different side chains as hole transport materials for perovskite solar cells. It has been shown that polymer **P3** containing triisopropylsilyl side groups exhibited better film formation properties and adhesion to perovskite absorber that, along with enhanced hole mobilities, resulted in the superior performance of devices. Encouraging PCE of 17.4% was achieved for non-doped **P3**-based PSCs that outperformed the efficiency of devices based on **P1** and **P2** with alkyl or alkoxyl substituents and benchmark **PTAA** polymer. Furthermore, we put forward that double-layered polymer films such as **P1**/**P3** or **P2**/**P3** might be promising HTMs due to the good ability of **P1** and **P2** for passivation of perovskite defects along with the good charge transport characteristics of **P3**. These findings feature the great potential of benzodithiophene-based conjugated polymers as dopant-free HTMs for the fabrication of efficient perovskite solar cells.

## Figures and Tables

**Table 1:** Physicochemical, optical, and electrochemical properties of polymers **P1**–**P3.**

	M_w_, kDa (M_w_/M_n_)	$\lambda_{max\;ABS\;}^{sol}$ $/\lambda_{max\;ABS\;}^{film}$ $/\lambda_{edge\;ABS\;}^{film},\;\mathrm{nm}$	$\lambda_{max\;PL\;}^{film},\;\mathrm{nm}$	$E_{gap\;}^{opt},\;\mathrm{eV}$	$E_{onset\;}^{ox},V\;\mathrm{vs}.\;\mathrm{Fc}/{\mathrm{Fc}}^{+}$	HOMO, eV	LUMO*, eV	T_d_, °C
**P1**	13.3 (2.3)	562/594,638/689	693	1.84	0.40	−5.20	−3.36	322
**P2**	14.7 (2.3)	572/587,632/683	678	1.85	0.39	−5.19	−3.34	313
**P3**	21.6 (1.8)	580,627/ 582,626/673	670	1.86	0.36	−5.16	−3.30	329

* LUMO = HOMO + $E_{gap\;}^{opt}$ (eV).

**Table 2:** Hole mobilities of **P1**–**P3** and photovoltaic performance of PSCs based on **P1**–**P3.**

HTM	μ_h_, cm^2^V^−1^s^−1^	V_OC_, mV	*J*_SC,_ mA cm^−2^	FF, %	PCE, %
**PTAA**	n/a	1020 * (1040 ± 14) **	20.9 (21.0 ± 0.9)	74 (68 ± 3)	15.7 (14.7 ± 0.6)
**P1**	3.63 × 10^−5^	1060 (1000 ± 51)	19.9 (21.1 ± 0.9)	65 (57 ± 4)	13.7 (12.2 ± 1.1)
**P2**	2.89 × 10^−5^	1000 (980 ± 12)	20.7 (18.7 ± 1.3)	60 (52 ± 6)	12.4 (9.7 ± 1.5)
**P3**	4.10 × 10^−5^	1080 (1060 ± 11)	20.9 (20.9 ± 0.5)	77 (74 ± 1)	17.4 (17.1 ± 0.6)

* maximal value (average ± standard deviation), ** average characteristics for sixteen devices.

## Data Availability

The data presented in this study are available in this article.

## Supplementary Materials

The following supporting information can be downloaded at: https://www.mdpi.com/article/10.3390/molecules27238333/s1, Figure S1: GPC chromatograms for polymers **P1**–**P3** measured against polystyrene standards; Figures S2–S4: ^1^H NMR spectra of polymers; Figure S5: FT-IR spectra of polymers **P1**–**P3**; Figure S6: DSC curves for polymers **P1**–**P3**; Figure S7: XRD patterns of thin films of conjugated polymers **P1**–**P3**; Figure S8: Absorption spectra of polymers **P1**–**P3** in 1,2-dichlorobenzene solution and thin films (a–c) and temperature- dependent UV-visible absorption spectra for **P1**–**P3** in 1,2-dichlorobenzene solution (d–f); Figure S9: SEM (top) and AFM (bottom) images for perovskite/HTMs bilayer stacks: MAPbI_3_/**P1** (a); MAPbI_3_/**P2** (b); MAPbI_3_/**P3** (c); Figure S10: Contact angle measurement of a water droplet of placed on the surface of the polymer films; Figure S11: Optical images of glass substrates covered with polymers **P1** (a), **P2** (b), and **P3** (c).
