# Supplementary material for: Novel Push-Pull Benzodithiophene-Containing Polymers as Hole-Transport Materials for Efficient Perovskite Solar Cells
Source: Molecules. 2022 Nov 29;27(23):8333. doi: 10.3390/molecules27238333 (PMC9741263; doi:10.3390/molecules27238333)
Supplement: Supplementary file 1 [file molecules-27-08333-s001.zip › molecules-2041302-supplementary.pdf]

molecules

MDPI

Supporting Information

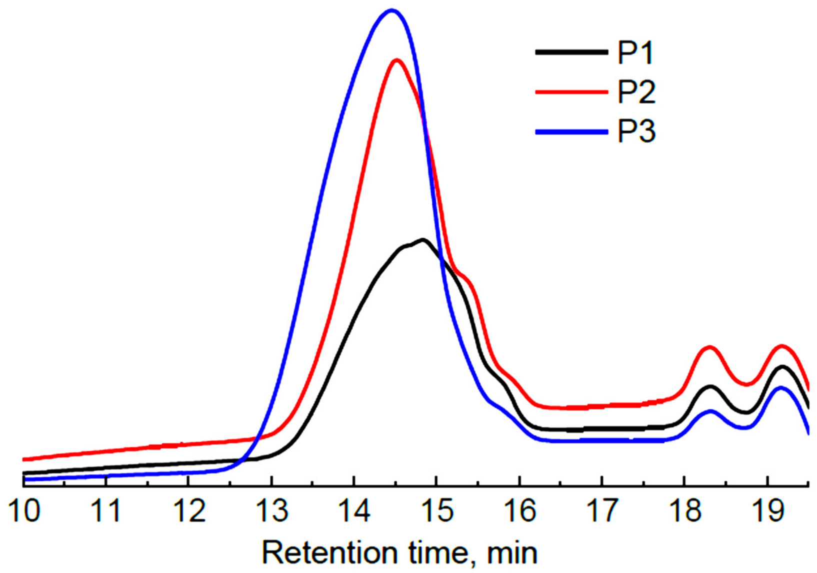

Figure S1. GPC chromatograms for polymers **P1-P3** measured against polystyrene standards.

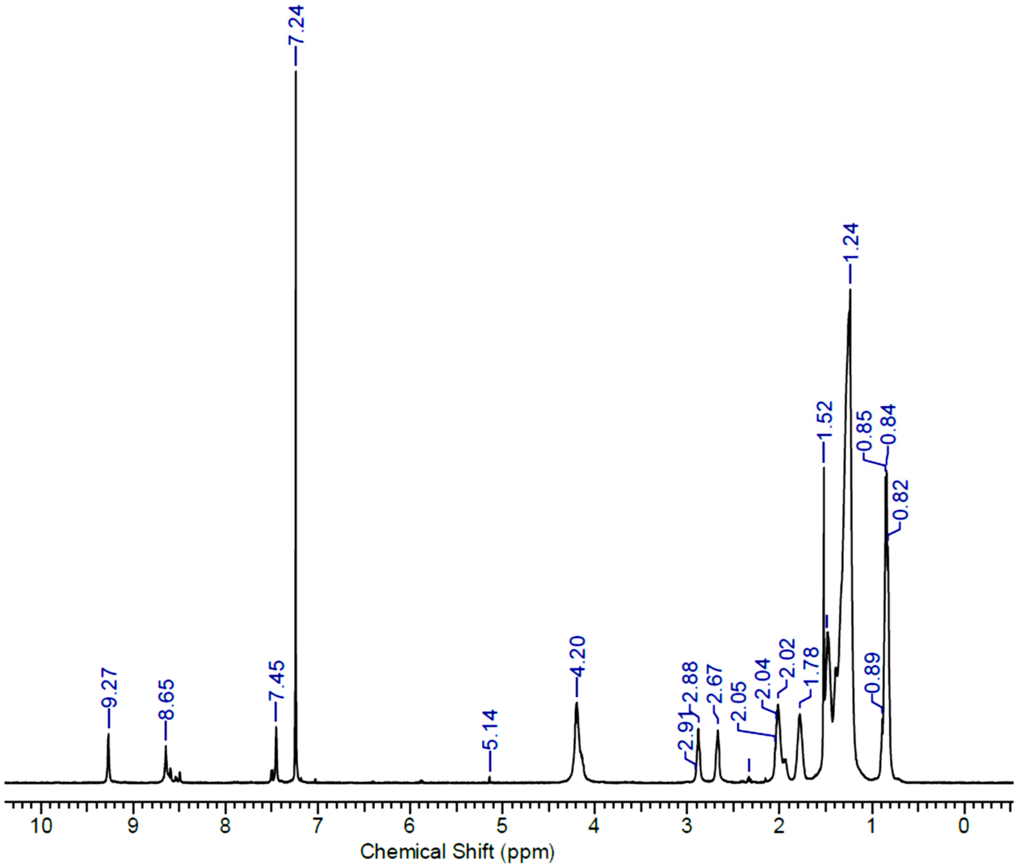

Figure S2. NMR spectrum of polymer **P1**

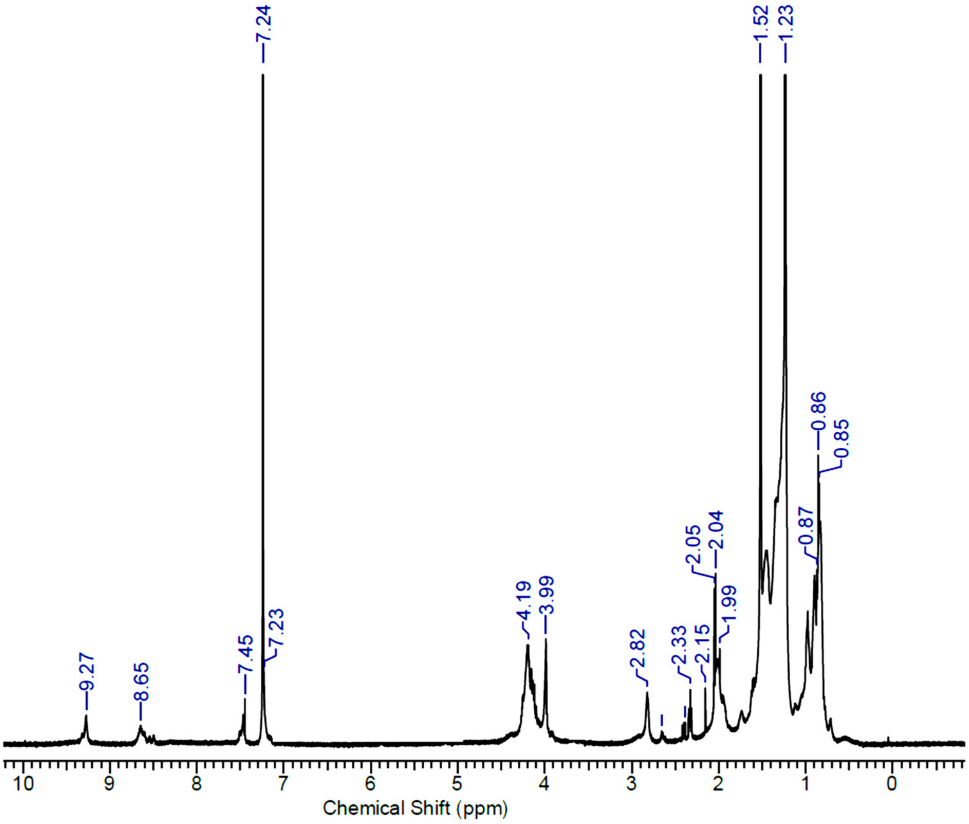

Figure S3. NMR spectrum of polymer **P2**

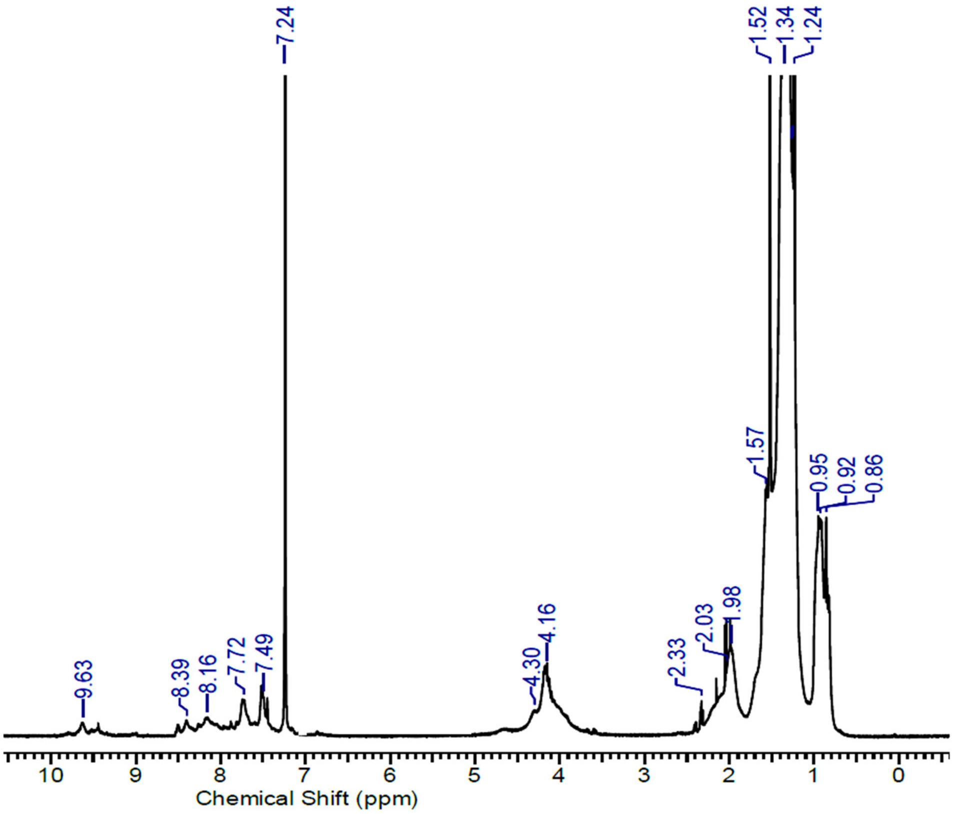

Figure S4. NMR spectrum of polymer **P3**

molecules

MDPI

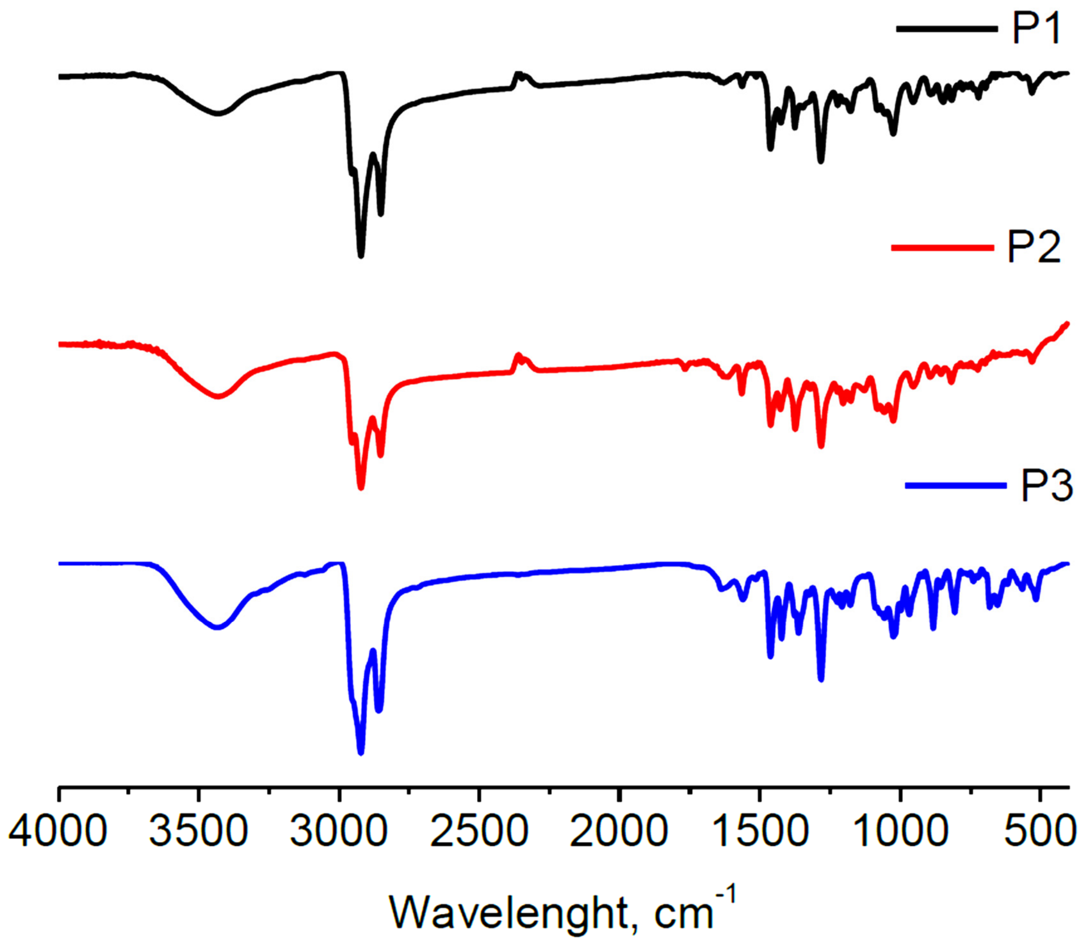

Figure S5. FT-IR spectra of polymers **P1**-**P3**

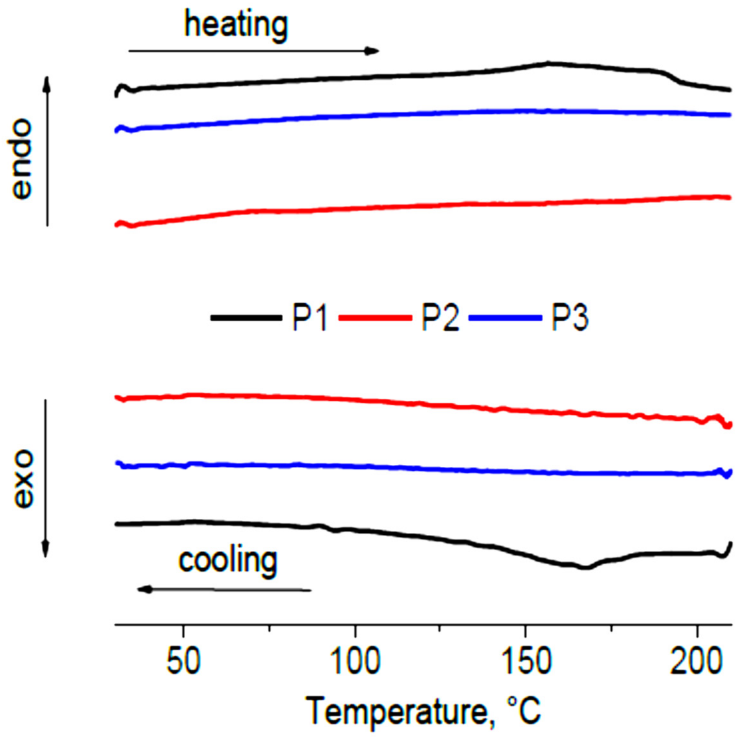

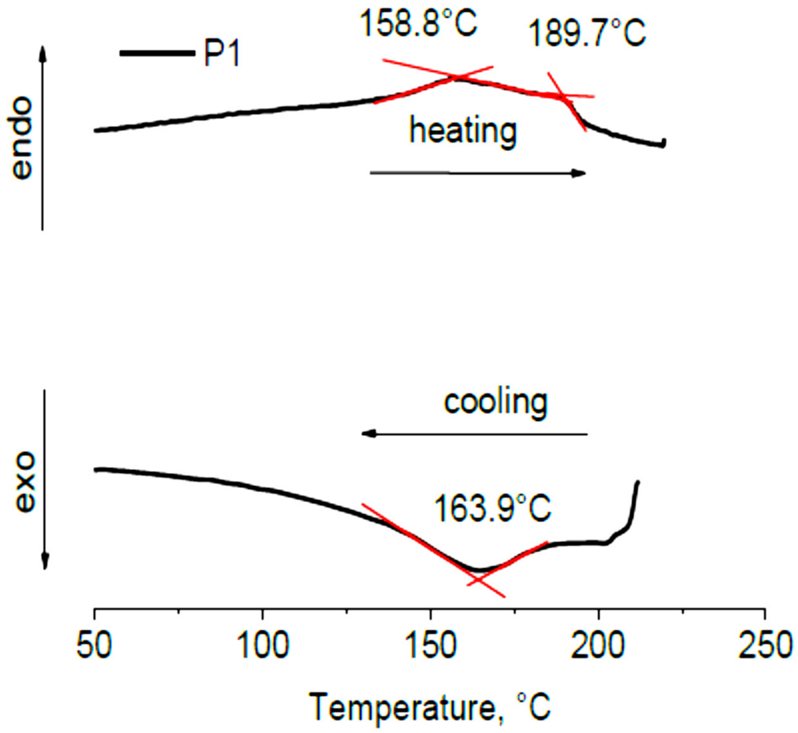

Figure S6. DSC curves for polymers **P1**-**P3** (N_2 flow 20 mL/min, heating rate of 10°C min^{-1}).

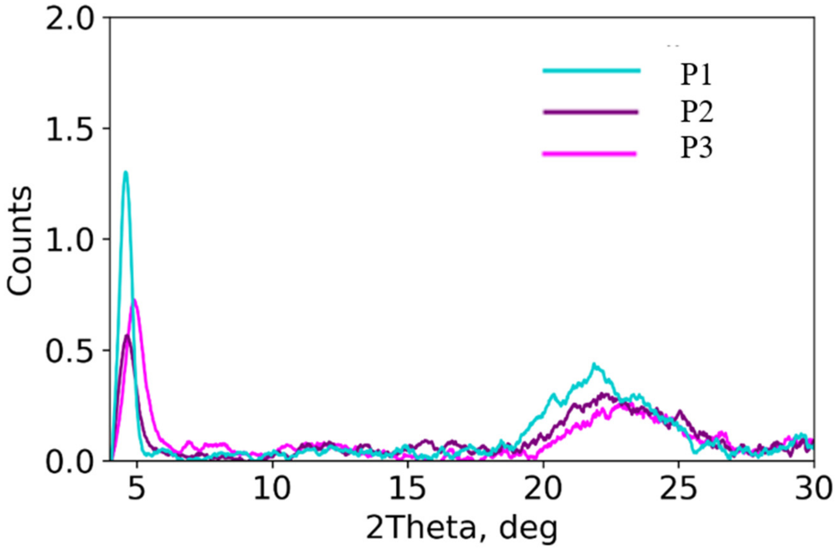

Figure S7. XRD patterns of thin films of conjugated polymers **P1**-**P3**

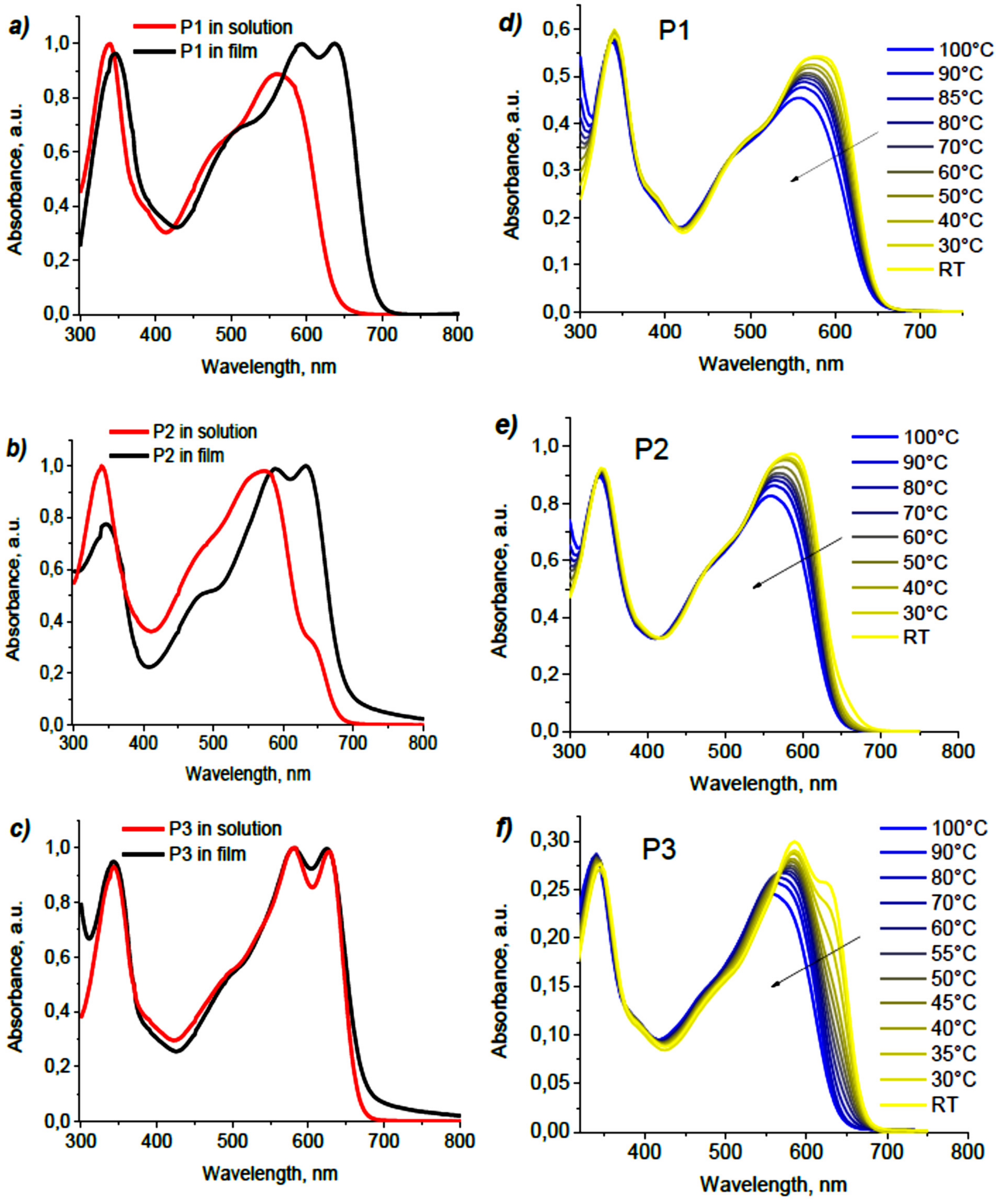

Figure S8. Absorption spectra of polymers **P1**-**P3** in 1.2-dichlorobenzene solution and thin films (a-c) and temperature-dependent UV-visible absorption spectra for **P1**-**P3** in 1.2-dichlorobenzene solution (d-f)

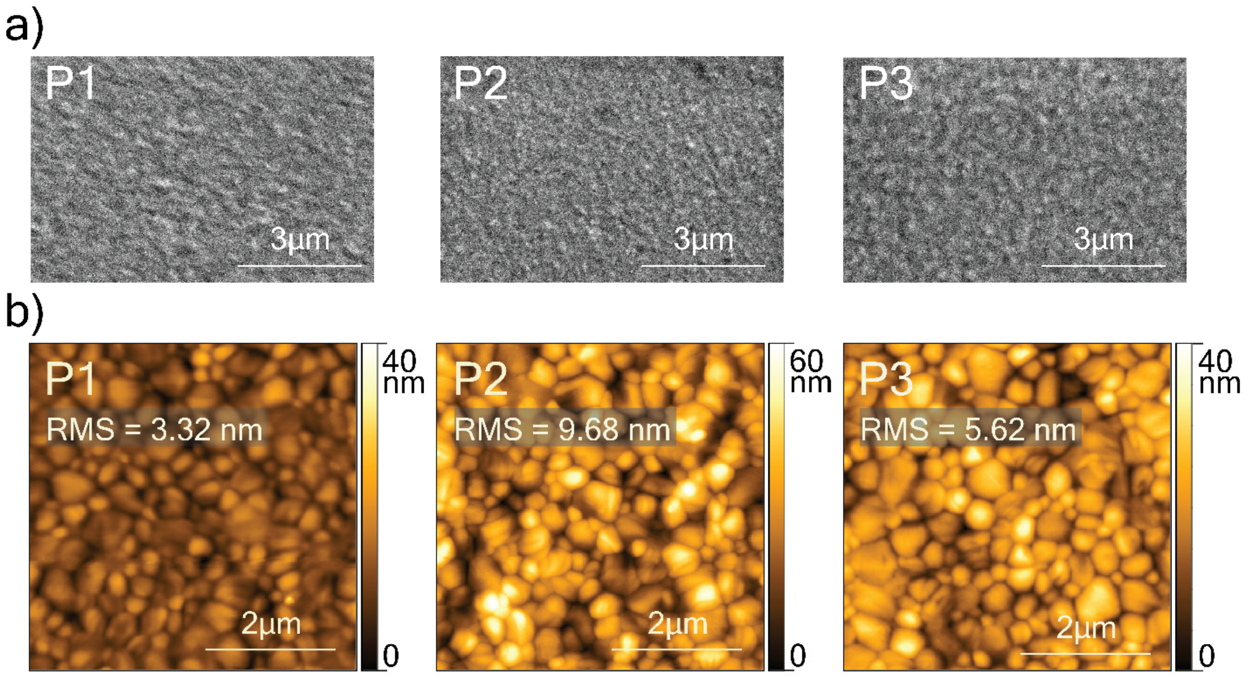

Figure S9. SEM (a) and AFM (b) images for perovskite/HTMs bilayer stacks.

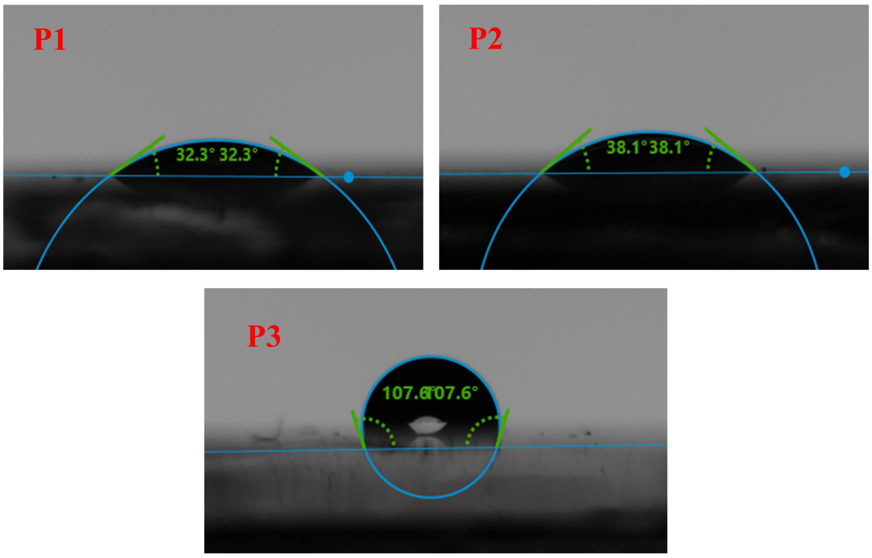

Figure S10. Contact angle measurement of a water droplet of placed on the surface of the polymer films

a)

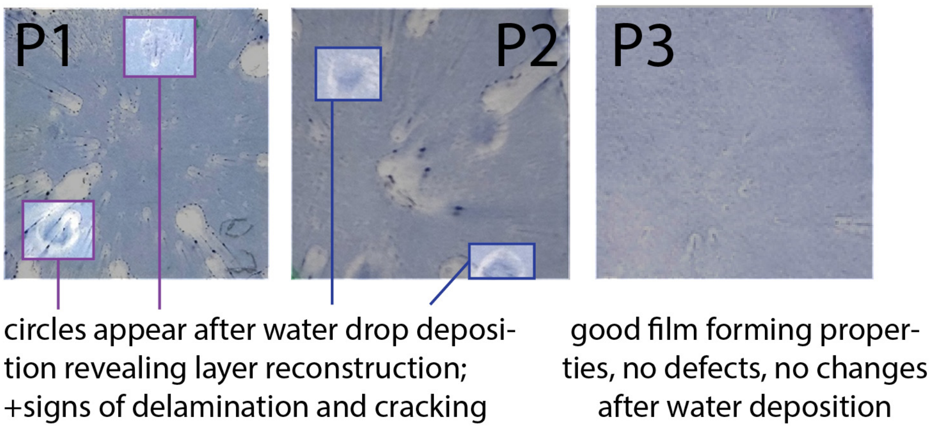

b)

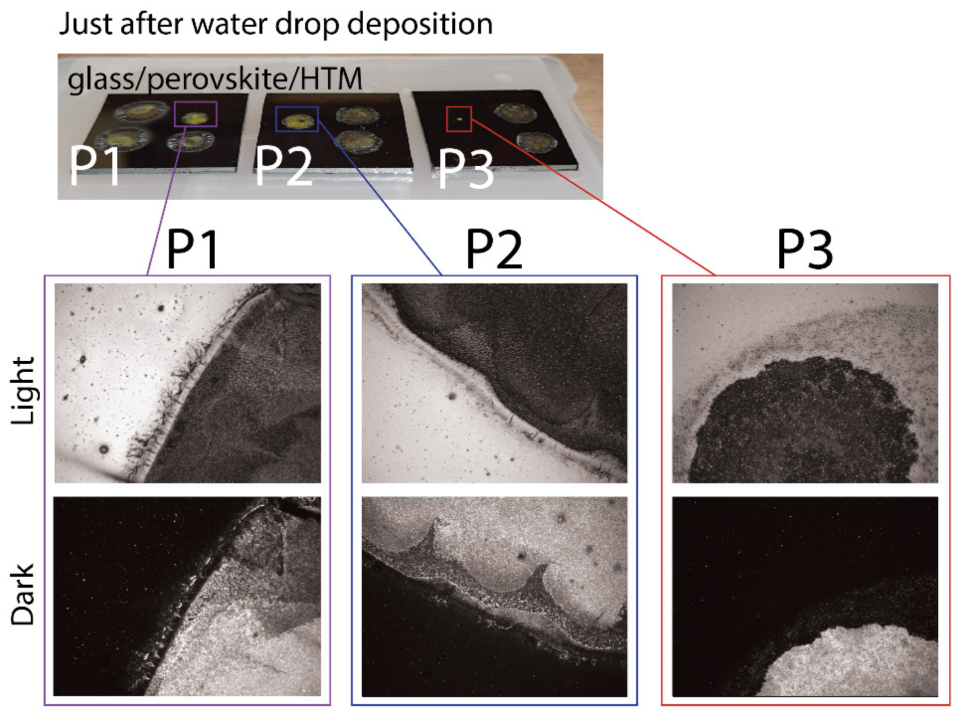

Figure S11. Optical images of glass substrates covered with polymers **P1** - **P3** (a) and optical microscopy images (b)
